# Efficiency of cold atmospheric plasma, cleaning powders and their combination for biofilm removal on two different titanium implant surfaces

**DOI:** 10.1007/s00784-021-04300-0

**Published:** 2022-01-05

**Authors:** Julia Kamionka, Rutger Matthes, Birte Holtfreter, Christiane Pink, Rabea Schlüter, Thomas von Woedtke, Thomas Kocher, Lukasz Jablonowski

**Affiliations:** 1 Department of Restorative Dentistry, Periodontology, Endodontology, Preventive Dentistry and Pedodontics, Dental School, University Medicine Greifswald, Rotgerberstr. 8, 17475 Greifswald, Germany; 2 Imaging Center of the Department of Biology, University of Greifswald, Greifswald, Germany; 3 Leibniz-Institute for Plasma Science and Technology E.V. (INP), Greifswald, Germany

**Keywords:** Biofilm, Cold atmospheric plasma, Dental implant, Powder, Surface treatment

## Abstract

**Objectives:**

Biofilm removal is the decisive factor for the control of peri-implantitis. Cold atmospheric pressure plasma (CAP) can become an effective aid due to its ability to destroy and to inactivate bacterial biofilm residues. This study evaluated the cleaning efficiency of CAP, and air-polishing with glycine (APG) or erythritol (APE) containing powders alone or in combination with CAP (APG + CAP, APE + CAP) on sandblasted/acid etched, and anodised titanium implant surface.

**Materials and methods:**

On respective titanium discs, a 7-day ex vivo human biofilm was grown. Afterwards, the samples were treated with CAP, APG, APE, APG + CAP, and APE + CAP. Sterile and untreated biofilm discs were used for verification. Directly after treatment and after 5 days of incubation in medium at 37 °C, samples were prepared for examination by fluorescence microscopy. The relative biofilm fluorescence was measured for quantitative analyses.

**Results:**

Air-polishing with or without CAP removed biofilms effectively. The combination of air-polishing with CAP showed the best cleaning results compared to single treatments, even on day 5. Immediately after treatment, APE + CAP showed insignificant higher cleansing efficiency than APG + CAP.

**Conclusions:**

CAP supports mechanical cleansing and disinfection to remove and inactivate microbial biofilm on implant surfaces significantly. Here, the type of the powder was not important. The highest cleansing results were obtained on sandblasted/etched surfaces.

Clinical relevance.

Microbial residuals impede wound healing and re-osseointegration after peri-implantitis treatment. Air-polishing treatment removes biofilms very effectively, but not completely. In combination with CAP, microbial free surfaces can be achieved. The tested treatment regime offers an advantage during treatment of peri-implantitis.

## Introduction

Dental implant installation is a very common and accepted treatment method to replace hopeless or missing teeth. The longevity of implants, however, may be jeopardized by peri-implantitis, which may ultimately lead to implant loss. The problem of non-predictability of successful peri-implantitis treatment represents one of the big challenges in dentistry. Since the aetiology of peri-implantitis is similar to periodontitis [1], the removal of the dysbiotic microbial biofilm from the exposed implant surface [2] is the cornerstone of peri-implantitis therapy. A multitude of surgical debridement methods have been described such as rinsing with citric acid, delmopinol, chlorhexidine, abrasion with gauze pellets soaked in saline and/or chlorhexidine, with air-powder devices, rotating or oscillating brushes or treatment with carbon dioxide or diode laser [3, 4]. Air powder devices exhibited the best cleansing capability of all mechanical methods. However, 20 to 60% of the exposed surface still remains untreated, depending on the treatment angle, even during optimal access, especially in the undercuts of the implant threads [5]. The microrough implant surface (roughness below 50 microns) and the implant threads provide “protected areas” for the microorganisms, inaccessible to conventional mechanical removal. In dogs or monkeys despite access-flap surgery the application of single decontamination measure, either chemical or mechanical, was not adequate to provide a satisfying healing resolution [3]. A literature review did not find any debridement method superior to any other in removing the biofilm and no method was able to achieve a stable result over time [6].

One disadvantage of all these therapeutic approaches is that not all bacterial deposits are removed [7]. Surfaces contaminated by microbes prevent healing and are not conducive to bone-forming cells. Therefore, surface decontamination is the critical step for resolution of the inflammation. The crucial role of biofilm removal has been illustrated in a dog study where instrumentation of smooth and rough implant surfaces with a curette and a saline soaked gauze pellet yielded inferior healing on rough compared to smooth implants, probably because cleaning was less efficient on rough surfaces compared to smooth surfaces [8]. These findings concur with long-term observations in patients [9].

A further option is the use of non-thermal cold atmospheric pressure plasma (CAP) as an adjunct to mechanical debridement. Cold plasma is an electrically neutral ionized gas, which includes charged particles (ions and electrons), an electric field, reactive species, and electromagnetic radiation in parts of vacuum ultraviolet, visible light and infrared spectrum (VUV/UV, VIS, IR) [10]. It can reduce the carbon content on treated surface [11] and thus also the microbes [12], has an antimicrobial effect [13], and generates a bone-conductive physico-chemical surface without damaging the surface of the implant [14].

Recently, we showed that the combination of a power-driven, nylon brush with subsequent CAP treatment led to a complete biofilm removal from titanium discs and enhanced osteoblastic cell spreading comparable to the sterile control discs [15]. However, at close inspection we could find nylon smears of the brush on the rough titanium sample, which was probably not conducive for long-term healing. In a subsequent study, we replaced the brush with an air polishing device with a nonabrasive powder and we showed that erythritol powder together with CAP has a high potential to render microbially contaminated implant surface cell compatible without microscopical damage of the rough titanium surface [16].

In all these studies we used sandblasted acid-etched discs. Since anodized surfaces have a completely different microstructure and since various animal as well a human studies pointed to, that these surfaces may favour progression of peri-implantitis [17–20], we wanted to find out, if air polishing in combination with CAP has a differential impact on removal of biofilm from anodized vs sand blasted acid etched (SLA) surfaces.

In our last experiment we did not find any difference in osteoblast spreading on discs treated only with air-polishing or with air-polishing and CAP [16]. We speculated that the use of an air polishing powder containing 0.3% chlorhexidine diacetate (CHX) besides erythritol was the reason for this successful surface decontamination. To determine if a CHX containing air polishing powder has more beneficial impact on plaque removal than a powder without CHX, we compared the erythritol (+ CHX) powder with a glycine air polishing powder on two different microrough titanium implant surfaces with or without additional CAP treatment. For this study, we hypothesised that (i) the powder with erythritol will show higher cleansing efficacy compared the treatment with glycine powder and (ii) the CAP treatment will compensate the difference between glycine and erythritol powder treatment resulting a high level of biofilm debridement and surface decontamination.

## Material and methods

### Titanium discs

We used titanium discs with an anodised (TiUnite, Nobel Biocare AB, Göteborg, Sweden) and a sandblasted and acid etched surface by the Discrete Crystalline Deposition process (BIOMET 3i, Zimmer Biomet, München, Germany) with a diameter of 5 mm, and thickness of 1 mm. The discs have an average roughness R_a_ at 612 ± 92.6 nm, an average roughness depth R_z_ at 2812 ± 331 nm for the anodised and a R_a_ at 2198 ± 222 nm, and R_z_ at 9406 ± 847 nm for the sandblasted/acid etched titanium surfaces (measured by Dektak 3 St Surface Profilometer, Irvine, CA, USA).

Before the samples with the anodised surface could be used, a small hole at the side of the specimen was sealed using a composite (Heraeus, Germersheim, Germany: Venus ® Diamond Flow Nano-Hybrid composite A3). Before use, the discs were cleaned in an ultrasonic bath and sterilised for 45 min at a temperature of 120 °C and pressure of 1 bar (Autoclave24, Melag, Berlin, Germany).

### Cultivation of biofilms

First, subgingival plaque was harvested from deep pockets of the same periodontally diseased volunteer for all tests. Plaque removal was approved by the ethics committee of the University Greifswald, medical department (Registration number: BB 120/10). The biofilm cultivation technique was previously described in detail [16]. In short, the discs were placed in 96-well micro titre plates (Techno Plastic Products AG, Trasadingen, Switzerland) and covered with 100 µl plaque. Biofilm was cultivated over 7 days at 37 °C after covering with Dulbecco`s Modified Eagle Medium (DMEM; Invitrogen GmbH, Karlsruhe, Germany) and the DMEM medium was renewed every 24 h. After culture, the medium was removed, and titanium discs were washed with 0.9% sodium chloride solution. Subsequently, the discs were placed into new sterile micro-titre plates.

### Treatment modalities

Five different treatment modalities were used to remove the biofilms on discs: air-polishing with powder containing glycine (APG), air-polishing with powder containing erythritol (APE), cold atmospheric pressure plasma (CAP), and the combination of each air-polishing powders with CAP (APG + CAP, APE + CAP).

**Fig. 1:**
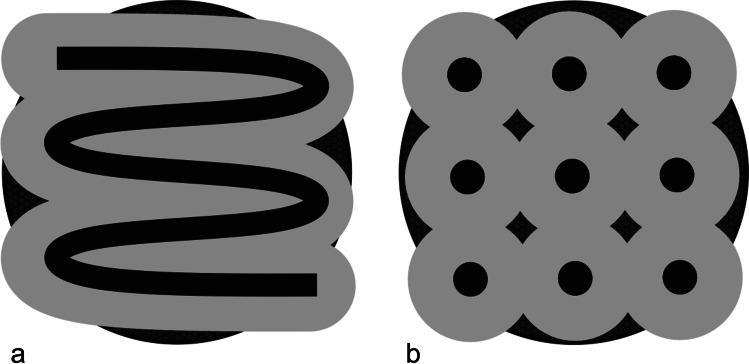
Sample processing: **a** mechanical treatment with Air-polishing (meander like). **b** CAP treatment (on 9 local points)

The air-polishing powders Air-Flow® Perio (based on glycine in particle size at 25 µm and amorphous silica < 10%) and Air-Flow® Plus (based on erythritol in particle size at 14 µm, 0.3% CHX and amorphous silica < 10%) (EMS, Nyon, Switzerland) were applied with EMS Master (EMS, Nyon, Switzerland), which was connected to the dental unit (air pressure 4.75 bar, water pressure 2.5 bar) with 4 units power setting and 100% water supply. The handpiece was fixed in a holder above the disc at a distance of 4 ± 1 mm at an angle of 65°. Both sides of the discs were treated first stationary at 4 prespecified spots for 15 s then in meandering motion for 30 s (Fig. 1a). Complete treatment time was 90 s per each disc side. After treatment, the discs were rinsed with 1 ml sterile NaCl (0.9%) and placed with the original biofilm covered side up on a sterile microtiter plate for further CAP treatment or analysis.

For CAP treatment, a plasma jet (kINPen® 08; Leibniz Institute for Plasma Science and Technology (INP), Greifswald, Germany) with a frequency of around 1 MHz at 2–6 kVpp and 3.5 W maximal input DC-power was used with the inert gas argon (99,999%; ALPHAGAZ; Air Liquide, Düsseldorf, Germany) as carrier gas with a gas flow of 5 slm (standard litre per minute) which was controlled by a flow controller (MKS Instruments, Munich, Germany) [21]. The length of the visible plasma plume was 10 mm and the temperature was between 63 and 46 °C, dependent on power input and axial distance from the capillary nozzle of the plasma source [22]. A computer-controlled x/y/z table (modified EDX-20; Roland DG, Westerlo, Belgium) directed the plasma jet at 9 spots and treatment was carried out for 60 s at each spot (Fig. 1b). Hence, the complete treatment time was 9 min for each disc side. The distance between the nozzle of the pen device and the disc surface was 5 mm during treatment. Finally, the sample was stored in a sterile microtiter plate with the last treated side up.

Untreated discs with biofilm (BF) served as negative control and sterile discs (ST) as positive control. Each treatment was carried out on anodised and sandblasted/acid etched titanium discs and were evaluated immediately after treatment (day 0) as well as after 5 days (day 5) of culture incubation at 37 °C in DMEM medium. The tests were repeated 5 times with 2 samples each (*n* = 10), for each test and control group (Σ *n* = 70) on the two different surfaces (Σ *n* = 140) (Table 1).

**Table 1:** Description of treatment groups, sample size, and examination times

	Day 0	Day 5	
Group	samples	samples	Treatment and controls
ST	2	2	Untreated sterile control
BF	2	2	Untreated biofilm control
APG	2	2	90 s air-polishing with glycine powder
APE	2	2	90 s air-polishing with erythritol powder
CAP	2	2	9 min cold atmospheric pressure plasma
APG + CAP	2	2	90 s air-polishing with glycine powder and 9 min cold atmospheric pressure plasma
APE + CAP	2	2	90 s air-polishing with erythritol powder and 9 min cold atmospheric pressure plasma
Each passage	14	14	Total: 28
5 repetitions	70	70	Total: 140

### Evaluation of biofilm removal by fluorescence microscopy

Samples (discs i.e. neg. ctrl, pos ctrl., test samples) were stained with 10 µM in distilled water diluted fluorescence dye Syto9® (Thermo Fisher Scientific, Invitrogen™, Schwerte, Germany) to label nucleic acids on day 0 and day 5. After staining, the discs were washed with 500 µl of distilled water during gentle movement of the microplate and dried in dark, before microscopy (BX 60, twofold magnification lens, GFP filter, Olympus, Hamburg, Germany). Digital images were taken using a camera (SLR; EOS 450D, Canon, Krefeld, Germany) with following adjustments: Program: M, Tv: 0.5 s, ISO: 200/24°, WB: Manually, jpg: L (large).

Finally, the resulting images were evaluated with the software “Image J” (v1.50, US National Institutes of Health, Bethesda, MD, USA). Therefore, a circular mask was used to enclose the visible disc and to analyse the grey values of recorded fluorescence signals. The relative biofilm fluorescence (BfF) value was determined for each test group based on image grey scale. Calculations for mathematical statistics: BfF = IntDen − (Disc area * Mean_Background); IntDen: Integral over the grey scale density, Area: Selected area for image analyse, Mean_Background: Mean grey level values of the sterile control). The BfF values are dimensionless and are only given as a number. Differences of the BfF values are given in percent.

**Fig. 2:**
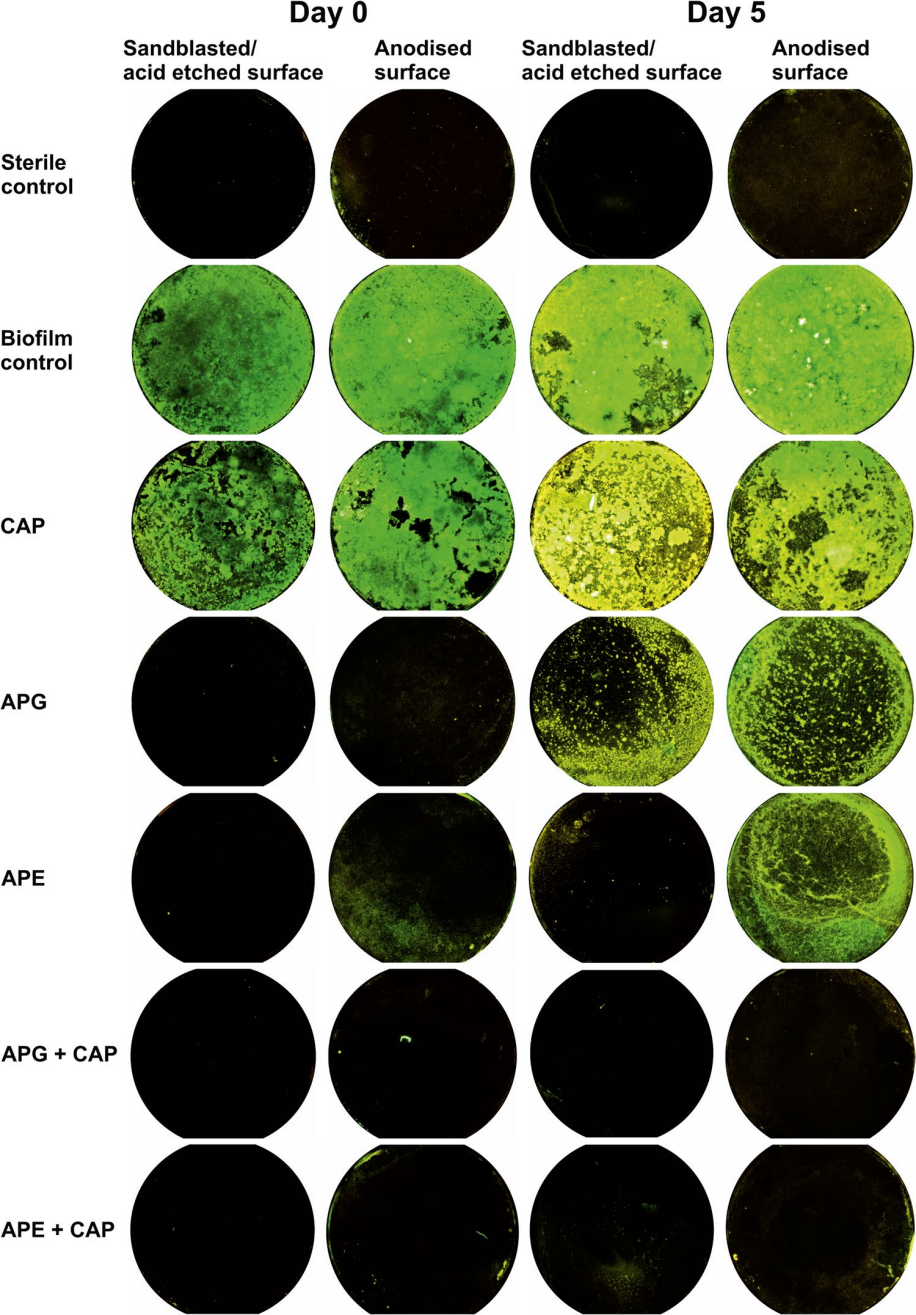
Fluorescence microscopy images of Syto9® stained biofilms on sandblasted/acid etched or anodised titanium discs of untreated control specimen (sterile and with biofilm), and specimen after treatment with plasma (CAP), air-polishing with powder containing glycine (APG), or erythritol (APE), and the combined treatment of APG + CAP, or APE + CAP directly (day 0) or after 5 days (day 5) of culture in medium

Figure 2 shows a visual impression of fluorescence levels between different treatments. The higher the value of the fluorescence, the more clearly the corresponding discs were coloured dominantly green.

### Scanning electron microscopy

The samples were fixed with 2.5% glutaraldehyde (SERVA Electrophoresis, Heidelberg, Germany) in buffered saline solution. Sample preparation for scanning electron microscopy and examination was carried out according to Kerlikowski et al. [23]. All micrographs were edited by using Adobe Photoshop CS6.

### Statistical analysis

**Fig. 3:**
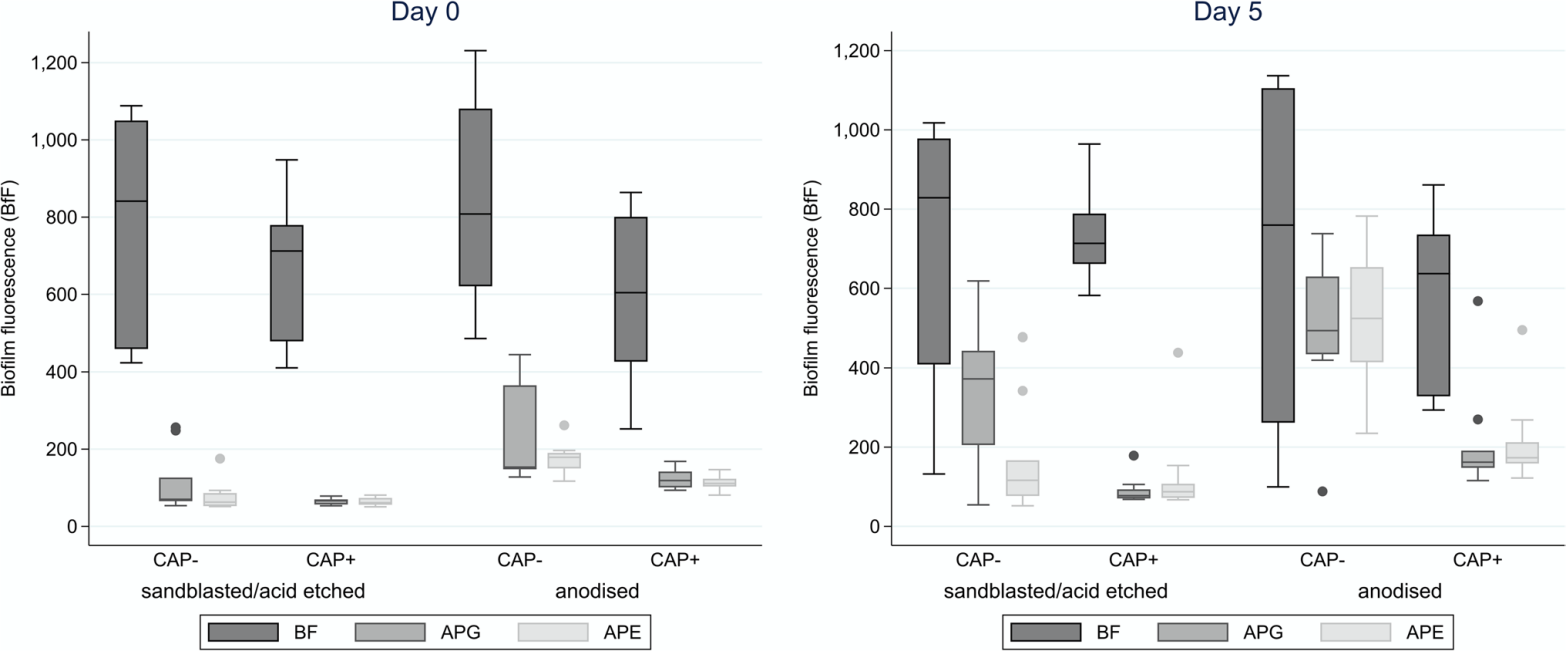
Relative biofilm fluorescence (BfF) values after treatment with APG, APE on sandblasted/acid etched or anodised titanium discs with or without subsequent CAP treatment (CAP + , CAP-) immediately (day 0) or after 5 days of culture in medium (day 5)

For the statistical analysis, 280 samples were evaluated (140 samples for both time points each). Relative biofilm fluorescence data (dimensionless quantity values of the grey tone histogram) were presented as medians with 25% and 75% quantiles. Boxplots were used to visualize distributional differences in biofilm fluorescence values by air-polishing powder (APG versus APE versus BF), titanium surface (sandblasted/acid etched versus anodised), and plasma application (CAP versus no CAP) (Fig. 3). Median regression models were applied to estimate effects of titanium surface, air-polishing powder, and plasma application on BfF values for both culture time points, day 0 and day 5 after treatment.

## Results

### Biofilm reduction

Both, in the sterile control as well in all powder treated groups the anodised titanium surface had about 2-time higher median values than the sandblasted/acid etched surfaces (Table 2).

**Table 2:** Relative biofilm fluorescence (BfF) values presented as median (25%; 75% quantile) after treatment with APG, APE on sandblasted/acid etched or anodised titanium discs with or without subsequent CAP treatment (CAP + , CAP-) directly (day 0) or after 5 days of culture in medium (day 5), and for the biofilm control (BF) as well sterile control (ST), reduction of BfF value regarding biofilm control in % (*Red. %*), *N* = 10 per group

Treatment and controls	Day 0				Day 5			
	Sandblasted/acid etched	*Red. %*	Anodised	*Red. %*	Sandblasted/acid etched	*Red. %*	Anodised	*Red. %*
BF	841.3 (459.4; 1050.0)		808.1 (620.8; 1080.9)		828.7 (408.7; 978.2)		759.5 (261.6;1105.2)	
CAP	712.1 (479.1; 779.5)	*15.4*	604.3 (426.4; 800.7)	*25.2*	713.5 (661.5; 788.6)	*13.9*	637.1 (328.3;735.7)	*16.1*
APG	70.0 (65.1; 126.5)	*91.7*	153.4 (147.7; 365.2)	*81.0*	372.2 (205.2; 443.2)	*55.1*	493.2 (434.8;629.2)	*35.1*
APE	62.7 (52.3; 86.3)	*92.5*	178.9 (150.0; 190.3)	*77.9*	116.1 (76.6; 166.8)	*86.0*	523.9 (414.3;653.6)	*31.0*
APG + CAP	66.3 (56.5; 69.8)	*92.1*	118.6 (100.7; 142.1)	*85.3*	77.5 (70.5; 93.4)	*90.6*	162.0 (147.6;191.2)	*78.7*
APE + CAP	61.1 (55.9; 73.7)	*92.7*	111.1 (103.0; 123.5)	*86.3*	87.4 (71.8; 107.6)	*89.5*	173.3 (158.6;212.4)	*77.2*
ST	60.5 (52.3; 64.8)	*92.8*	162.8 (127.6; 188.9)	*79.9*	64.4 (57.4; 68.0)	*92.2*	224.2 (167.7; 230.0)	*70.5*

Data are presented as median (25%; 75% quantile) million gray scale values

Day 0: The sole CAP treatment lowered minimally the biofilm fluorescence values on both surfaces compared to the biofilm control surfaces, about 15.4 and 25.2% (Table 2). Irrespective of surface, both airflow powder treatments without and with additional CAP achieved fluorescence values between 77.9 to 92.7% lower compared to biofilm controls. In comparison to the sterile controls, which showed a fluorescence of about 20% for anodised etched surfaces and 7% on sandblasted/acid etched surfaces, the BfF values were higher on anodised surfaces (Appendix Table 4). Biofilm fluorescence values did not differ significantly according to powder type, while additional CAP treatment slightly increased biofilm fluorescence values. However, for both titanium surfaces, all treatment methods achieved significantly different BfF values compared to the untreated biofilm control (*p* < 0.001) (Table 3). Differences in biofilm fluorescence values between treatment methods and between both titanium surfaces were not statistically significant (*p* = 0.6, Table 3).

**Table 3:** Results for median regression models of relative biofilm fluorescence values on titanium surface, air-polishing powder, plasma application and their multiplicative interactions (*p* for interaction < 0.10)

	Day 0		Day 5	
	*Β* (95% CI)	*P*	*Β* (95% CI)	*P*
Titanium surface (ref. sandblasted/acid etched)				
Anodised surface	63.73 (− 1.60; 129.06)	0.056	226.97 (120.32; 333.61)	< 0.001
Air-polishing powder (ref. BF)				
APG	− 774.23 (− 887.38; − 661.07)	< 0.001	− 393.15 (− 523.76; − 262.53)	< 0.001
APE	− 776.68 (− 889.84; − 663.53)	< 0.001	− 518.57 (− 649.19; − 387.96)	< 0.001
CAP treatment (ref. CAP-)				
CAP +	− 294.78 (− 407.94; − 181.63)	< 0.001	− 44.45 (− 195.27; 106.37)	0.56
Interaction between Air-polishing powder and CAP treatment				
APG × CAP +	270.62 (110.59; 430.65)	0.001	− 186.65 (− 371.36; − 1.93)	0.048
APE × CAP +	265.94 (105.92; 425.97)	0.001	− 45.16 (− 229.88; 139.55)	0.629
Interaction between titanium surface and CAP treatment +				
Anodised surface × CAP +	-	-	− 152.68 (− 303.50; − 1.86)	0.047

*B*, median regression coefficient; *CI*, confidence interval; *BF*, untreated biofilm control; *APG/APE*, air-polishing with glycine/erythritol containing powders; *CAP* + */CAP-*, cold atmospheric pressure plasma (used/not used)

Day 5: Biofilm fluorescence was reassessed after a 5-day recultivation period of microbial residues. On anodised discs, BfF values rebounded from about 160 at day 0 to 500 (ca. 46%), irrespective of the powder used. In contrast, on sandblasted/acid etched surfaces the rebound to BfF values raised comparably lower from about 70.0 to 372.2 (36.6%), after APG or from 62.7 to 116.1 (6.6%) after APE treatment (Table 2), which shows additionally an interesting discrepancy of BfF values between the two powders with a fluorescence reduction of 30.9%. Irrespective of the titanium surface, both powders in combination with CAP application resulted in BfF values comparable to corresponding values of the sterile control surface (Table 2).

The BfF values after day 0 and day 5 are visualised in boxplots of Fig. 3.

## Discussion

**Fig. 4:**
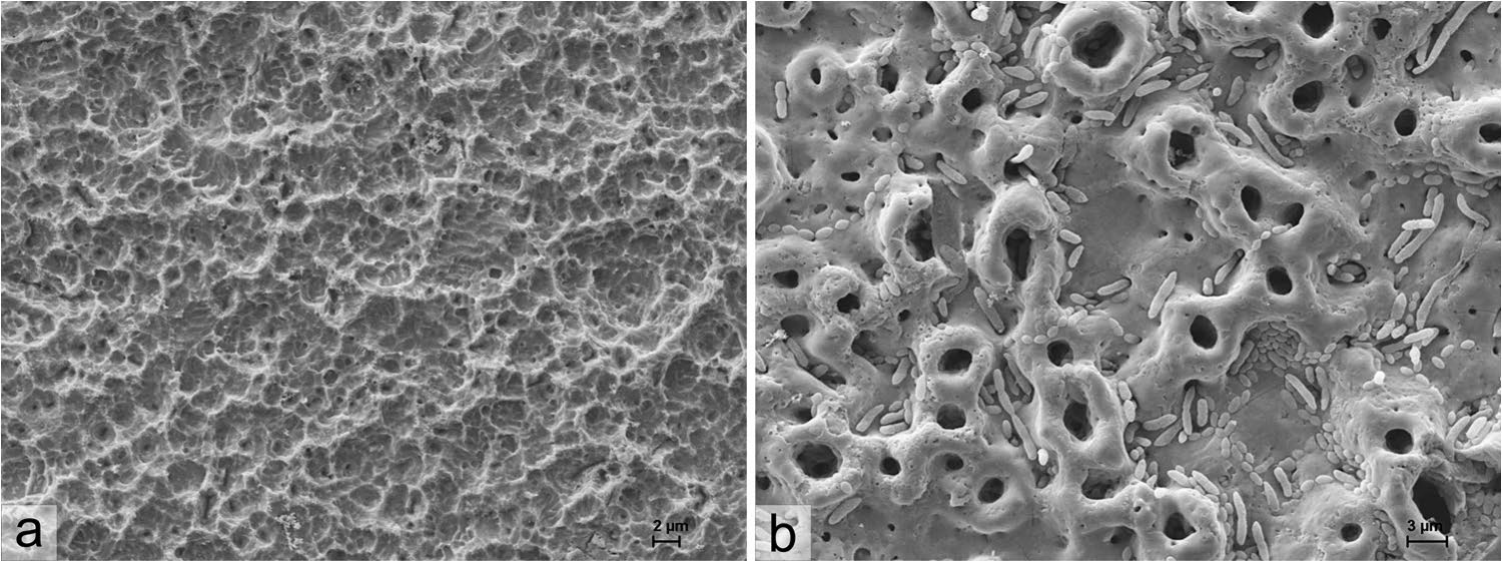
Scanning electron micrographs of air-polishing with powder containing erythritol (APE) treated biofilms on **a** sandblasted/acid etched or **b** anodised titanium discs as an example for the different surface structures and an impression of cleansing results. Evaluation was carried out after 5 days (day 5) of cultivation in medium

A key goal in peri-implantitis therapy would be the complete removal of biofilm from the exposed implant surfaces without any changes of the native implant macro- and micro-surface structure. In this study, different treatments were tested (APG, APE, CAP, APG + CAP, APE + CAP) on two different customary titanium implant surfaces, sandblasted/acid etched, and anodised microstructure. The best biofilm reductions were obtained with the combination of air-polishing and CAP on the sandblasted/acid etched surface, which became apparent after 5 days of cultivating (day 5). Investigation only on day 0 was insufficient to determine the effectiveness of the cleaning methods because while the remaining microorganisms were not visible, they appeared to be present and able to proliferate when grown for 5 days. Thus, the success in anodised surface is not quite as good as with sandblasted/acid etched surface because the implant structure of this anodised surface has more undercut areas and holes where microorganisms are more protected against mechanical cleansing (Fig. 4). The interaction of air-polishing with CAP in these areas seems to be helpful to generate surfaces where no microbial regrowth was visible. Simple surfaces such as sandblasted/acid etched can also be cleaned well only with powder [16].

The severity and progression of peri-implantitis are pronounced differently depending on the implant surface [24]. As shown before, plasma alone has nearly no cleaning performance that was in concordance with previous studies [15, 16], while in combination with air-polishing, almost the entire biofilm could be removed. Powder seems to allow more efficient cleaning than a brush, as demonstrated by Duske et al. [15], because the microbial regrowth was slightly more extensive, but was happen for both powders used. Air-polishing treatment on anodised surface showing slightly better success with APG. For sandblasted/acid etched surface, the difference between the powder types was not significant, but a tendency that APE is more effective than APG is recognisable, which can be caused by smaller powder particles (Ø APE 14 µm, APG 25 µm), because smaller powder particles could achieve smaller niches of the titanium structure, or by the additional chlorhexidine content that can inhibit microbial regrowth after treatment [25, 26]. Although Hägi et al. [27] demonstrated that APG showed higher abrasiveness compared to APE. Sterile discs were obtained after combined treatment of plasma and powder.

The results in our work suggest a benefit in the treatment methodology, by the combination of plasma and air-polishing, because in addition to its ability to remove biofilm [28] CAP also acts as an antimicrobial agent [13, 29].

CAP represents a promising complement to conventional cleaning methods. Shi et al. was able to show in a study on beagles that in ligature-induced peri-implantitis a significantly higher bone level can be obtained by treatment with plasma than without after treatment with chlorhexidine but not in combination with a mechanical treatment [30].

However, the success of the biofilm removal and inactivation of this study cannot fully predict the outcome in clinical setting or under clinical conditions. The patient’s plaque biofilm was grown in vitro, the specimens had a simple geometry, the accessibility to treat the biofilms on surface was ideal, and a sterile environment was possible in laboratory but would not be feasible in oral cavity. Further experiments should incorporate investigations on implants in clinical practice-relevant model and aspects of biocompatibility of the treated surfaces have to be evaluated.

## Conclusion

The purpose of this study was to show whether the removal of biofilm on two different implant surfaces (sandblasted and acid etched, anodised) can be improved by CAP after air-polishing with glycine or erythritol + chlorhexidine powders. Evaluated after 5 days of incubation after treatment, neither a sole CAP nor a sole air-polishing treatment can completely remove and inactivate the microorganisms from all surfaces tested, but a combination of air-polishing with subsequent CAP application led to biofilm-free and seemingly sterile surfaces. The structure of the implant surface was a significant factor, i.e., the results on sandblasted/acid etched surface were better than those on anodised surface. Since CAP has an antimicrobial effect and achieves biofilm reduction, it is a promising therapeutic option for the treatment of peri-implantitis.

## Appendix

Table 4

**Table 4:** Relative biofilm fluorescence (BfF) values presented as median after treatment with APG, APE on sandblasted/acid etched or anodised titanium discs with or without subsequent CAP treatment (CAP + , CAP-) directly (day 0) or after 5 days of culture in medium (day 5), and for the biofilm control (BF) as well sterile control (ST), ratio of BfF values to biofilm control in % (*% of BF*)

Treatment	Day 0				Day 5			
	Sandblasted/acid etched	*% of BF*	Anodised	*% of BF*	Sandblasted/acid etched	*% of BF*	Anodised	*% of BF*
BF	841.3		808.1		828.7		759.5	
CAP	712.1	*84.6*	604.3	*74.8*	713.5	*86.1*	637.1	*83.9*
APG	70.0	*8.3*	153.4	*19.0*	372.2	*44.9*	493.2	*64.9*
APE	62.7	*7.5*	178.9	*22.1*	116.1	*14.0*	523.9	*69.0*
APG + CAP	66.3	*7.9*	118.6	*14.7*	77.5	*9.4*	162.0	*21.3*
APE + CAP	61.1	*7.3*	111.1	*13.7*	87.4	*10.5*	173.3	*22.8*
ST	60.5	*7.2*	162.8	*20.1*	64.4	*7.8*	224.2	*29.5*
